# Testing a workplace physical activity intervention: a cluster randomized controlled trial

**DOI:** 10.1186/1479-5868-8-29

**Published:** 2011-04-11

**Authors:** Rosemary RC McEachan, Rebecca J Lawton, Cath Jackson, Mark Conner, David M Meads, Robert M West

**Affiliations:** 1 Yorkshire Quality and Safety Research Group, Bradford Institute for Health Research, Bradford Teaching Hospitals NHS Foundation Trust, Bradford, UK; 2 Institute for Psychological Sciences, University of Leeds, Leeds, UK; 3 School of Healthcare, University of Leeds, Leeds, UK; 4 Academic Unit of Health Economics, Leeds Institute of Health Sciences, University of Leeds, Leeds, UK; 5 Division of Biostatistics, Leeds Institute of Genetics, Health and Therapeutics, Leeds, University of Leeds

## Abstract

**Background:**

Increased physical activity levels benefit both an individuals' health and productivity at work. The purpose of the current study was to explore the impact and cost-effectiveness of a workplace physical activity intervention designed to increase physical activity levels.

**Methods:**

A total of 1260 participants from 44 UK worksites (based within 5 organizations) were recruited to a cluster randomized controlled trial with worksites randomly allocated to an intervention or control condition. Measurement of physical activity and other variables occurred at baseline, and at 0 months, 3 months and 9 months post-intervention. Health outcomes were measured during a 30 minute health check conducted in worksites at baseline and 9 months post intervention. The intervention consisted of a 3 month tool-kit of activities targeting components of the Theory of Planned Behavior, delivered in-house by nominated facilitators. Self-reported physical activity (measured using the IPAQ short-form) and health outcomes were assessed.

**Results and discussion:**

Multilevel modelling found no significant effect of the intervention on MET minutes of activity (from the IPAQ) at any of the follow-up time points controlling for baseline activity. However, the intervention did significantly reduce systolic blood pressure (B = -1.79 mm/Hg) and resting heart rate (B = -2.08 beats) and significantly increased body mass index (B = .18 units) compared to control. The intervention was found not to be cost-effective, however the substantial variability round this estimate suggested that further research is warranted.

**Conclusions:**

The current study found mixed support for this worksite physical activity intervention. The paper discusses some of the tensions involved in conducting rigorous evaluations of large-scale randomized controlled trials in real-world settings.

**Trial registration:**

Current controlled trials ISRCTN08807396

## Background

There is now convincing evidence that people who are physically active live longer and have lower morbidity than those who are inactive [1-3]. It is recommended that adults engage in 30 minutes of at least moderate intensity activity on at least five days of the week [4]. In North America, however, less than half the population are meeting the recommended levels of physical activity, and this is lower still in the UK: 28% (women) to 40% (men) [5-7]. Moreover, a reduction in the manufacturing industry and a rise in more sedentary jobs such as those in the service industry across the western world means that individuals are sedentary for a large proportion of the day; a risk factor for chronic disease [8]. The workplace is a useful setting in which to promote physical activity (either by encouraging physical activity during the working day or in leisure time), since most adults spend half their waking hours at work. Moreover, the potential economic benefits to an organization such as reduced absenteeism, increased productivity, increased stress tolerance and improved decision-making, as well as the physical and mental health benefits for employees, means that there is a strong business case for using the workplace as a vehicle for health promotion efforts of this kind [9].

Current evidence as to the effectiveness of workplace physical activity interventions has been mixed. Meta-analytic reviews have found that workplace physical activity interventions have small positive effects on self-reported physical activity (*d *= 0.11 to 0.26) [10-12], and varying effectiveness for fitness (e.g. *d *= 0.15, [10]; *d *= 0.47-0.57, [12]). Moreover, variation in findings by outcome measurement is a feature of these studies. Some measure physical activity through self-report measure or via pedometer; the latter being problematic as wearing a pedometer can serve to increase activity without any other intervention [13,14]. Other studies employ health outcomes such as blood pressure [15], girth and heart rate [16,17]. In addition, very few workplace studies perform an economic analysis to explore the cost-effectiveness of interventions. Generally, it is recognized that there is a need for more methodologically robust studies that take into account issues of randomization and blinding [17], and assess behavior change over longer follow-up periods [10]. It is generally recognized that any intervention should be based upon explicit theory [18-20].

The current study addresses these limitations by assessing the effects of the theory based AME (Awareness, Motivation, Environment) for ACTIVITY intervention [20] on both self-reported physical activity and objective indicators of health. An economic cost-benefit analysis is also performed.

The AME for ACTIVITY intervention is based on the Theory of Planned Behavior [21], and was developed using an intervention mapping approach [19]. The Theory of Planned Behavior (TPB), states that behavior is determined by intentions (motivation) toward engaging in the behavior and actual control over the behavior (which can be split into self-efficacy and perceived control) [22]. Intentions, in turn are determined by attitudes toward engaging the behavior, social norms and perceived behavioral control. Attitudes capture the overall evaluation of the behavior and include both an affective (the extent to which the behavior is seen as enjoyable) and an instrumental (the extent to which the behavior is seen as beneficial) component [23]. Social norms refer to the perceptions of social pressure to engage in the behavior and encompass both injunctive norms (e.g. perceptions of what important others think) and descriptive norms (e.g. perceptions of what important others actually do) [23]. The model has been found to typically account for between 41-46% of the variance in physical activity intentions and 24-36% of the variance in behavior [24-26]. Further detail on the intervention can be found within the method section and a full description of its development is available elsewhere [20].

In summary, the aim of the current project was to evaluate the effect of the AME for ACTIVITY intervention in improving both self-reported physical activity and objective measures of health assessed over a 12-month period amongst employees from different organizations. An economic analysis aimed to explore whether the intervention was cost-effective. A matched pairs cluster randomized control trial design was employed, with department (worksite) as the unit of allocation, in order to minimise potential contamination amongst intervention and control employees. Data were analysed using multi-level modelling clustering time-points within individuals, and individuals within worksites, with results reported at the individual level. We hypothesized that intervention participants would engage in significantly more physical activity over the 12 month follow-up than control participants, and exhibit improvements in objective measures of health.

## Methods

### Design

A matched-pairs cluster randomized controlled trial was used. Pairs of worksites, matched according to function and size were randomly assigned to intervention or control by the first author using a random number generator. Participants were not explicitly told which group they were in, although true blinding was not possible as intervention activities were noticeable in intervention worksites. Measures of physical activity were taken at baseline (T1), 3 months (0 months post-intervention, T2), 6 months (3 months post-intervention, T3) and 12 months (9 months post intervention, T4). Health data were collected (see measures) at baseline and 12 months. We set out to detect a standardized effect size of 0.2 on physical activity levels based on previous reviews [10,11], using a two-sided significance level of 0.05 and minimum power of 0.80. Thus a total sample size of 902 (451 in each group) was required. We assumed that participants within the same workplace (cluster) would be fairly independent; thus sample size calculations were based on an intra-cluster correlation of 0.01. The study was funded by the BUPA foundation medical charity (reference BUPAF/33a/05).

### Participants

Five organizations were approached (Bus Company; Hospital; Local Government Council; National Government Organization; University) according to a purposive sampling frame to ensure wide representation of different occupations. All agreed to take part in the study. Forty-four eligible worksites (i.e. those which could be matched with a similar worksite in the same organization in a different geographical location, e.g. two bus depots) were recruited via local contacts, and 4349 employees were invited to take part. In order for the intervention to be acceptable to all organizations, an inclusion criterion was that all employees would be eligible to take part unless they were excluded on the basis of the following medical criteria (compiled by a consultant respiratory physician). Eighty-six employees were excluded on this basis.

• Known heart disease requiring medication use (e.g. angina, previous heart attack) or permanent pace maker.

• Significant valvular heart disease (e.g. aortic or mitral valve disease, or a heart murmur)

• On medication that alters heart rate (e.g. beta blockers, calcium channel blockers or digoxin)

• Significant breathing problems including asthma of a level that makes it difficult to exercise or climb a flight of stairs in one go

• Have had chest pain within the previous four weeks

• Have had a heart attack, angina, heart surgery within the previous three months

• Pregnant

**Figure 1:**
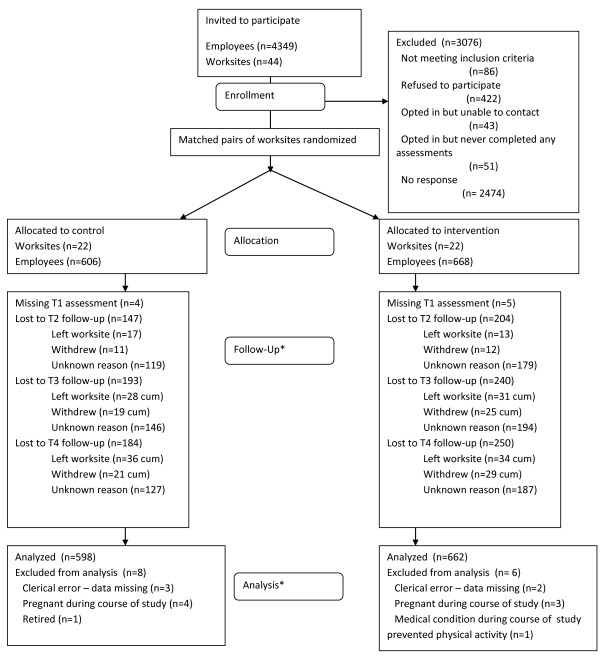
Consort flow diagram for participants and worksite (* all 44 worksites remained in the study, cum = cumulative).

The total sample comprised 1260 individuals who provided measurement for at least one time point (only 9 of which did not provide data at baseline), from 44 worksites. Eighty-six respondents were excluded. Figure 1 shows the CONSORT flow diagram for participants throughout the study.

### The intervention

The intervention took the form of an easy to implement toolkit, delivered in-house by trained local facilitators (volunteer employees with no specialist skills or knowledge) over a three-month period. The objective of the intervention was to increase levels of at least moderate intensity activity (in at least 10 minute bouts with a view to achieving the current recommended levels of at least 30 minutes on at least 5 days of the week) [3,4,27,28]. Employees were encouraged to be more physically activity either during the day (for example in lunch breaks) or in leisure time. The intervention development process was informed by a literature review, focus groups and a detailed intervention mapping process [19] and is described elsewhere [20]. The intervention targeted the theoretical constructs from the Theory of Planned Behavior (affective and instrumental attitude, injunctive and descriptive norms, self-efficacy and perceived control, intention). Briefly, we first identified our specific outcomes (e.g. increase of moderate intensity activity in work or leisure time), and performance objectives (the specific steps employees would have to engage in to achieve the outcomes, e.g. express confidence in managing competing demands [at work/in leisure time]). We then translated these into change objectives which explicitly specified the change required in each of the theoretical constructs. For each change objective we selected appropriate theoretical methods (e.g. for self-efficacy: enactment, modelling cf. Bandura) and translated them into practical strategies (e.g. role model stories [modelling], successful management of competing demands [enactment]). Finally we created an organized program plan selecting components and strategies which were feasible for delivery within the current project.

The final intervention consisted of 8 key components, and a launch week. The components were: a knowledge quiz, interactive leaflets, posters, team challenges, reminders, letters of management support, newsletters, and fridge magnets to allow self-monitoring of physical activity. Thus the intervention was delivered at both the worksite (e.g. management support, team challenges) and individual level (e.g. interactive leaflets, self-monitoring of activity).

Each month of the intervention had a different theme allowing different messages about the beneficial effects of physical activity to be conveyed. Month 1 focused on the physical health benefits of physical activity, month 2 on mental health benefits (e.g. reduced stress) and month 3 on the social benefits (e.g. spending time with family and friends).

A timetable was given to facilitators which advised which components were to be delivered when. For example, in week 1 of the intervention facilitators were instructed to 'launch' the intervention, distribute the first of 3 interactive leaflets, display relevant posters, distribute the self-monitoring fridge magnet and letter of management support, and run a 'knowledge' quiz. In week 2 they were asked to run a physical activity 'team challenge', in week 3 they were asked to circulate a reminder message about the benefits of activity, and in week 4 they were asked to distribute a newsletter highlighting that months activities. Subsequent months followed a similar structure with some activity happening each week. A timetable can be found in Additional file 1 - suggested timetable.

All materials were supplied to the facilitators, along with a manual instructing facilitators what should be done and when. The intervention was designed to be flexible to meet the needs of different organizations, for example facilitators could choose different types of team challenges to run depending on their worksite (for example, team based activities for those working in offices, or individual improvement challenges for those working individually - e.g. bus drivers). As a guide, if implemented as instructed to groups of up to 50 individuals, the intervention would take approximately 15 hours of facilitator time over a three-month period.

The intervention had a clear visual identity and logo (see http://www.psyc.leeds.ac.uk/ameforactivity/). The components of the intervention were coded by two independent raters according to Abraham and Michie's [29] taxonomy of behavior change techniques. The key techniques employed in descending order of focus were; providing information about health benefits (apparent in 8/8 components) and consequences (7/8), engendering social support/social change (7/8), prompting intention formation (5/8), time management (5/8), prompting specific goal setting (4/8), rewards (4/8), using prompts and cues (4/8), providing instruction (3/8), reviewing behavioral goals (3/8), self-monitoring (3/8), feedback (2/8), behavioral contract (leaflets only) and role modelling (leaflets only). See Additional file 2 - behavior change techniques.

The intervention was usually delivered by 1-2 local facilitators in each worksite (1 per 20-30 employees - 15 worksites used 1 facilitator, 6 used two and one large worksite of 100 employees used 5). Facilitators were volunteer employees with no specialist skills or knowledge and were nominated by managers or supervisors within each of the organizations. Each facilitator received one days training and the intervention manual. A member of the research team phoned the facilitators each month to gain feedback on implementation of the intervention and record which components were being delivered. Across the 22 intervention worksites a median of 6.4 components were delivered (interquartile range 4 - 8).

### Procedure

Ethical approval for this study was granted by Institute of Psychological Sciences, University of Leeds and Sheffield East NHS local research ethics committee in October 2007. Pairs of worksites were recruited to the study between October 2007 and May 2008, and followed up for 12 months. Employees within worksites were sent personalized invitation letters (with the exception of four worksites that were unable to provide names of employees due to confidentiality concerns). Each employee who opted into the study was then contacted by a member of the research team and their eligibility assessed according to the exclusion criteria. Eligible employees were then sent the first questionnaire and a health check appointment was arranged (see below for further details). Health check dates for each of the matched pairs of worksites were scheduled on successive days. At the time of the health check, control participants received a brief leaflet describing ways of improving health through diet and activity. This leaflet was also given to intervention participants.

On completion of health checks at a particular worksite all intervention materials were dispatched and facilitators set a launch date for the intervention within two weeks. The second questionnaire was sent out immediately post intervention for each matched pair of worksites. Questionnaire reminders were then sent at 3 weeks and 5 weeks after the original questionnaire. This same reminder procedure was utilized for all subsequent questionnaires. All questionnaires were anonymous and participants' data were matched using an anonymous code.

### Measures

#### Primary outcome measure: Moderate - Vigorous MET minutes of Physical Activity

The primary outcome measure was the total MET minutes of moderate and vigorous physical activity accrued over the past 7 days. Since we were interested in a global measure of vigorous and moderate intensity activity we selected the short form of the International Physical Activity Questionnaire. This measure has demonstrated validity and reliability and performs similarly to the longer version of the questionnaire [30,31]. It exhibits moderate correlations with objectively assessed physical activity via pedometer or accelerometer data [31-34], performing similarly to other questionnaire physical activity indices [32]. The IPAQ short form continues to be used in a number of physical activity intervention studies [35-38].

#### Secondary outcome measures

##### Objective measures of health and fitness

Objective measures of health and fitness were obtained for respondents who participated in the health checks (1215 at Time 1; 612 at Time 4). Due to scheduling and resource constraints health technicians were only available at each worksite for a set number of days. This meant we were not able to offer everyone a health check at Time 2 as not all respondents had availability on the times and dates we were in their worksite. Opportunities to engage in the health check were on a first come, first serve basis. The health check took place in the participants' worksite and was conducted by a trained health technician who was blinded to participant condition. The following were assessed: percentage body fat and body mass index (using OMRON BF306 body fat monitor), diastolic and systolic blood pressure (lowest of two measurements using OMRON M7 blood pressure monitor), and resting heart rate (OMRON M7).

#### Other questionnaires

Demographic details including age, gender, and socio-economic status using the self-coded UK National Statistics Socio-Economic Measure (NS-SEC) [39] were recorded. Health-related quality of life was measured using the EQ-5D [40]. This measure includes a global rating of current health using a visual analogue scale ranging from 0 (worst imaginable) to 100 (best imaginable), referred to here as 'health score'. Other measures were also assessed but not reported here.

### Analysis

All analyses are reported at the level of the individual. The impact of the intervention on all outcomes was analysed using multi-level modelling in Stata Intercooled (version 10.0) controlling for age, gender, time-point (controlling for past behavior or other baseline measurements), NS-SEC social class, health score (0-100) and season of measurement. To assess the impact of the intervention we included a condition (intervention vs. control) variable; all baseline measurements were coded as control in recognition of the fact that at that point no-one had received the intervention. Individuals and worksites were set as random effects.

For all analyses time-points (4 for self-reported physical activity and 2 for objective measures of health) were nested within individuals, who were nested within worksites. The log ratio likelihood test for analyses with the primary outcome measure confirmed that this three-level structure was more appropriate than a 2-level structure (e.g. time-points within individuals, χ^2^(1) = 24.0, *p *< .001). Analyses were conducted in December 2009.

### Cost-effectiveness analysis

A societal perspective was adopted, accounting for direct costs to the health service and indirect costs and benefits to society. Costs of developing and delivering the intervention were collated by recording the amout of time spent developing the intervention by the research team, the amount of time delivering the intervention by the local facilitator (and costed using the relevant employment grade pay rates). These were combined with productivity changes calculated from self-reported absence due to ill-health [41] (costed using adjusted median UK earnings [42] and reduced to 80% [43]) and the opportunity cost of time engaged in physical activity. The opportunity cost to the individual was calculated by multiplying the number of hours spent in physical activity (based on Time 4 self reports) by the adjusted wage rate. However, to maintain a conservative view these costs were not reduced to 80%. Benefit was in terms of Quality Adjusted Life Years (based on EQ-5D utility valued using the UK General Population Tariff [44]) gained. The EQ-5D is the most commonly used instrument in QALY analyses and preferred by NICE as the basis for calculating QALYs [40,45]:

The resulting value for each individual was then used in a multi-level model to ascertain the cost-effectiveness of the intervention as a whole, as per the primary effectiveness analysis. The workplace effect was very small and the model including workplace failed to converge. Therefore we estimated a two level model including individuals and time-points only. The study had a 12-month follow-up period and therefore discounting was not required. We used the NICE cost effectiveness threshold of £20,000 per QALY to convert the mean incremental QALY (mean expected utility in the intervention group minus mean expected utility in the control group), into the Incremental Monetary Benefit. The incremental net benefit was calculated by subtracting the mean cost of the intervention from Incremental Monetary Benefit. Bootstrapping (500 simulations) was used to produce 95% confidence intervals on the Expected Net Benefit.

## Results

### Description of sample

The current sample was predominantly classified as 'White-British', were married or living with their partner, had children and were in the upper two categories of the NS-SEC. Table 1 shows that intervention and control groups were well matched (for interested readers baseline characteristics split by cluster can be found in Additional file 3- [cluster characteristics at baseline]), and the groups did not differ in self-reported MET minutes of moderate - vigorous activity at baseline (*t *= 0.237, df = 1031, *p *= n/s). Across the entire sample, 39.3% met the recommended guidelines of engaging in ≥150 minutes of at least moderate intensity activity over the past week at baseline, in line with UK population statistics. Characteristics of drop-outs at Time 4 compared with baseline were explored for both questionnaire responses and health check non-attendance using independent samples t-tests (continuous variables) or chi-square analysis (categorical variables). Compared with baseline, participants who did not return the questionnaire were more likely to report poorer health status at baseline (67.10 vs. 69.71, t = -2.36, df = 1088, p = 0.018), and were less likely to be in the upper socio-economic group (71.4% vs. 81.0%, ×2 = 12.75, df = 1, p < .001). There were no differences according to sex, age, intervention group or physical activity level at baseline.

**Table 1:** Description of sample at baseline

	Control (N = 598)	Intervention (N = 662)
% Male^a^	46.8% (N = 278)	45.2% (N = 296)
Age in years^b ^(SD)	42.46 (10.77)	43.13 (10.41)
Ethnicity^c^		
White British	90.5% (N = 496)	88.9% (N = 538)
White Other	4.2% (N = 23)	4.6% (N = 28)
Marital status^d^		
Married	56.9% (N = 313)	58.2% (N = 385)
Living with partner	17.5% (N = 96)	14.0% (N = 84)
In a relationship	8.0% (N = 44)	8.3% (N = 50)
Single	17.6% (N = 97)	13.6% (N = 82)
Children^e^		
None	36.4% (N = 200)	32.6% (N = 196)
1-3	59.4% (N = 326)	62.3% (N = 375)
4+	4.2% (N = 23)	5.1% (N = 31)
Highest educational qualification^f^		
Undergraduate or postgraduate qualification	46.5% (N = 238)	41.7% (N = 240)
Vocational qualification	20.9% (N = 107)	21.7% (N = 125)
School level qualification	32.7% (N = 167)	36.6% (N = 211)
NS-SEC^g^		
1. Managerial and professional	60.1% (N = 318)	58.7% (N = 343)
2. Intermediate	16.8% (N = 89)	20.4% (N = 119)
3. Small employers and own account workers	0	0
4. Lower supervisory and technical occupations	5.9% (N = 31)	5.7% (N = 33)
5. Semi routine and routine occupations	17.2% (N = 91)	15.2% (N = 89)
Organization		
Council (20 worksites)	42.3% (N = 253)	39.4% (N = 261)
Teaching Hospital (14 worksites)	18.4% (N = 110)	20.8% (N = 138)
Bus Company (4 worksites)	17.9% (N = 107)	18.9% (N = 125)
Government organization (2 worksites)	13.9% (N = 83)	13.4% (N = 89)
University (4 worksites)	7.5% (N = 45)	7.4% (N = 49)
% Meeting recommended guidelines (≥150 moderate - vigorous minutes per week)	39.8%	39.0%
Baseline MET minutes Vigorous/Moderate intensity	1124.02 (1753.51)	1098.80 (1662.08)
	*(N = 485)*	*(N = 548)*
HEALTH MEASURES		
Lowest systolic blood pressure	122.67 (15.69)	123.24 (16.10)
Lowest diastolic blood pressure	79.57 (10.31)	79.54 (10.68)
Resting heart rate	71.14 (69.53)	71.62 (11.27)
Percentage body fat	31.36 (7.69)	31.74 (7.83)
Body mass index	25.96 (4.67)	26.18 (5.20)

^a^11 missing cases; ^b^12 missing cases, standard deviation in parentheses; ^c^107 Missing cases; ^d^109 Missing cases; ^e^109 Missing cases; ^f^172 Missing cases; ^g^147 Missing cases, as only large organizations were approached there were no small employers or own account workers within the sample; ^h^91 Missing cases; standard deviation in parentheses.

A subset of respondents attended the Time 4 health check. Non attenders were significantly more likely to be male (*χ^2 ^*= 11.52, df = 1, p = .001), older (43.44 years vs. 42.13 years, t = 2.16, df = 1204 = 2.16, p = .031), less likely to be in the highest social class (69.3% vs. 86.2%, *χ^2 ^*= 39.17, df = 1, p < .001), marginally less likely to be in the intervention group (55.7% vs. 49.2, *χ^2 ^*= 4.25, df = 1, p = .039), and report a poorer 'current health state' at Time 1 (67.28 vs. 70.31, t = 2.95, df = 1056, p = .003). However no differences were apparent on self-reported moderate and vigorous MET minutes of physical activity at Time 1. With regards to health indices, drop-outs exhibited higher systolic (124 vs. 121 mmHg, t = 3.48, df = 1212, p = .001), diastolic (80.52 vs.78.55 mmHg, t = 3.28, df = 1212, p = .001) blood pressure and body mass index (26.85 vs. 25.31, t = 5.43, df = 1204, p < .001). No differences were apparent for baseline percentage body fat. Based on these differences social class, health status, were entered as control variables in all subsequent analysis. Baseline health indices were controlled for in the analyses with health indices as the outcome measure.

The intra-cluster correlation coefficient for the primary outcome measure was 0.05 indicating individuals within worksites were fairly independent of one another with regard to their levels of physical activity. A list of mean values and standard deviations for each of the outcome measures at each time point can be found in Additional File 4 - outcome measures at baseline.

### Primary outcome measure: physical activity

The results of the primary outcome analysis were based on 1025 respondents (from 44 worksites), who provided data on each variable in the model at between one and four time points (average = 2.8; see Table 2). The effect of the intervention on self-reported moderate/vigorous physical activity, controlling for past physical activity (along with other control variables, see below) was positive although non-significant (*B *= 52.70, 95%CI: -132.92 to 238.32).

**Table 2:** Primary outcome measure: MET Minutes moderate - vigorous physical activity

Variable	B	SE	Z	p	95% lo	95% hi
Age	2.48	4.26	0.58	.560	-5.87	10.83
Female^a^	-382.83	102.02	-3.75	.000	-582.80	-182.86
Intermediate^b^	168.02	121.79	1.38	.168	-70.62	406.78
Lower supervisory and technical^b^	790.52	227.36	3.48	.001	44.90	1236.13
Semi-routine and routine^b^	492.33	186.17	2.64	.008	127.46	875.22
Health score	18.46	2.01	9.17	.000	14.51	22.41
T2^c^	14.73	95.82	0.15	.878	-173.06	202.54
T3^c^	91.86	89.53	1.03	.305	-83.64	267.34
T4^c^	23.88	83.29	0.29	.774	-139.38	187.14
Spring^d^	263.10	75.41	1.48	.138	-36.10	260.80
Summer^d^	436.27	99.64	4.38	.000	240.97	631.56
Autumn^d^	112.35	75.74	1.48	.138	-36.10	260.80
Intervention	52.70	94.71	0.56	.578	-132.92	238.32
Constant	-310.86	252.28	-1.23	.218	-805.32	183.61

^a^Compared to males

^b^Compared to managerial and professional occupations

^c^Compared to baseline measurement (Time 1)

^d^Compared to winter

Physical activity did vary as a function of control variables (see Table 2). The lower social classes reported more physical activity than those in higher social classes due, at least in part, to those in higher social classes tending to occupy more desk-based sedentary jobs. Second, as health status improved the amount of self-reported physical activity increased. Third, participants reported more physical activity per week in summer compared with winter. Finally, women reported significantly less MET minutes moderate/vigorous activity than men. These results are not discussed further. In order to explore whether the intervention was more successful for those with lower initial physical activity we repeated the analysis using a past behavior × intervention interaction. No significant effect was found.

### Secondary outcome measure: Objective health measures

All analyses were based on 1029 individuals (from 44 worksites) completing at least one of the two health checks (average 1.5). Multi-level modelling controlling for seasonality, social class, age, health score, gender and baseline scores revealed no significant effect of the intervention on diastolic blood pressure or percentage body fat (results not reported). However, we did find significant effects of the intervention on systolic blood pressure, resting heart rate and body mass index. In the interests of parsimony significant effects of control variables are not reported, tables of the full analyses can be requested from the first author.

Controlling for all other variables, intervention participants showed significantly lower levels of systolic blood pressure than controls (-1.79 mmHg; 95%CI = -3.10 to -0.47), and lower resting heart rate; intervention participants exhibiting a value 2.08 beats less than the controls (95%CI: -3.28 to -.089). Finally, an intervention effect was apparent for BMI, indicating that the intervention increased BMI by 0.18 units (95%CI: 0.01 to 0.34) compared to the control group.

### Cost-effectiveness of the intervention

The estimated cost of developing the intervention was £22,809 (Table 3). The total cost of delivering the intervention per employee (including development) was £24. Costs of subsequent use of the intervention would be reduced as the development costs have already been incurred. Table 4 displays the mean utility and mean cost for the control and intervention groups, and the incremental net benefit for the workplace exercise intervention. The observed incremental net benefit was -£103.02 indicating the intervention did not benefit participating worksites. Using a non-parametric bootstrap we estimated the 95% confidence interval for the expected incremental net benefit to be -£4961.72 to +£4748.04.

**Table 3:** Costs of the intervention

Cost	Amount
**Development costs**	
Labour	£20,500
Equipment (e.g. Computers and printers)	£500
Consumables	£38
Travel	£21
Graphic design	£1,750
**Total**	£22,809
**Delivery costs**	
Labour	£13,253
Equipment (computers, exercise equipment)	£338
Graphic Design	£1,750
Prizes	£100
**Total**	£15,441
**Overall total cost**	£38,250
Average cost per participant (n = 662)	£58
Opportunity cost of physical exercise^a^	£5
Adjusted impact on productivity^b^	-£39

**Total overall cost per participant**	£24

^a^Difference in mean annual cost of physical activity; intervention vs control groups

^b^Difference in mean annual cost of sickness absence; (intervention vs control groups)*0.8

**Table 4:** Cost-utility analyses

	Control	Intervention
Mean intervention cost (£)	£0	£24
Mean Utility (SE)	0.899 (0.003)	0.895 (0.004)
Monetary Health Benefit (SE) ^a^	£17979.4 (59.13)	£17900.0 (86.93)
Net Monetary Benefit (SE) ^b^	£17979.4 (59.13)	£17876.4 (86.93)
Incremental Net Benefit^c^		-£103.02

^a^Monetary Health Beneft = Utility*20000

^b^Net Monetary Benefit = Monetary Health Benefit -Cost

^c^Incremental Net Beneft = Net Monetary Benefit Intervention - Net Monetary Beneft Control

## Discussion

The current study found mixed support for the intervention. We predicted that this worksite physical activity intervention would increase self-reported levels of physical activity compared to a control group and that this in turn would be associated with reductions in blood pressure, percentage body fat, resting heart rate and BMI. Whilst there was no change in self-reported physical activity, diastolic blood pressure or percentage body fat, the intervention group showed significantly greater reductions in systolic blood pressure and resting heart rate than the control group. In addition, BMI increased modestly in the intervention group compared with control.

The primary outcome for this study was self-reported physical activity. However, we failed to identify a significant impact of the intervention on this outcome. This is an important finding, particularly within the context of the significant effects for health outcomes related directly to increases in physical activity (resting heart rate and systolic blood pressure discussed below). One feasible explanation, that has wider implications for the evaluation of physical activity interventions, is that the self-report measures are not sufficiently sensitive to detect increases in PA over the longer term in trials of this kind. The validity of these measures depends on the ability of respondents to accurately recall all the different aspects of physical activity they have performed [46], as well as the extent to which they respond honestly [47]. Moreover, although one of the most widely used self-report measures of physical activity, the IPAQ has a tendency towards over-reporting (perhaps due to its' asking for average times and best estimates of frequencies [48]). It is possible that for those in the control group, who may not have been closely monitoring their physical activity levels, this over-reporting tendency is exaggerated. The problems of self-report measures are further supported by recent evidence that objectively-measured moderate and vigorous physical activity levels are more strongly associated with health outcomes than self-reported measures [49], and that self-report measures can lead to underestimations of energy expenditure [50]. Indeed, the error estimates associated with these instruments means that some have argued that they are not valid when making individual level estimates of physical activity [51].

In this trial we also found that the intervention significantly reduced systolic blood pressure. This is encouraging, particularly in light of evidence that suggests systolic blood pressure has greater significance than diastolic blood pressure for cardiovascular risk, particularly in later life [52,53]. Reductions in systolic blood pressure of 2 mm Hg (similar to the levels found here) are associated with around 10% lower stroke mortality and 7% lower mortality from ischemic heart disease or other vascular causes in middle aged populations [54]. The intervention compared to control also showed a reduction in resting heart rate, an important health outcome because higher resting heart rates are associated with the risk of coronary events in both men [55,56] and women [57].

Moreover, there is convincing evidence that these greater decreases in systolic blood pressure and resting heart rate found among intervention participants were a direct result of them being more physically active. For example, recent evidence suggests that moderate levels of physical activity are most commonly associated with changes in systolic rather than diastolic blood pressure [58-60]; and resting heart rate has also been identified as a variable that is associated with changes in physical activity during intervention programs [61]. However, the effect of the intervention on these two indices in the absence of changes in self-reported physical activity is puzzling, not least because a meta-analysis of worksite physical activity interventions demonstrated stronger effects for self-reported activity than for fitness measures [10]. However, a recent meta-analysis of worksite physical activity interventions coded for length of follow-up of data collection after the intervention was completed [62] identified only six RCTs that employed follow-up periods longer than 6 months. None of these studies employed both fitness/health and self-reported measures of physical activity studies. One possible explanation for these findings is that the health measures employed here show greater sensitivity to change for measurement over an extended period of time. In other words, the health measures might better reflect the activity levels of the sample across the 12 months when compared to the physical activity measures that represent a snapshot of activity over the last week

The impact of the intervention on body mass index is at first counter-intuitive. There has been recent criticism of the emphasis on body weight (and hence BMI) as an outcome for physical activity interventions. For example, in a recent study investigating the impact of an intensive exercise program for 58 overweight/obese men and women, 26 failed to show the predicted weight loss given their energy expenditure [63]. These individuals did however show reductions in blood pressure (particularly systolic blood pressure) and resting heart rate. In a meta-analysis of school based interventions, 15 of the 18 studies showed no effect of physical activity interventions on BMI [64]. This suggests that interventions which aim to increase levels of moderate/vigorous activity should not necessarily expect to see associated changes in BMI and body fat. It is possible, of course, that participants in such studies compensate for higher levels of activity by consuming more calories; or that they increase muscle mass, something we did not measure here.

Whilst the economic evaluation indicated that the current intervention was not effective, there is substantial uncertainty (as evidenced by the large 95% confidence intervals) around this estimate suggesting further research is warranted. In addition, the cost-utility results may have been compromised due to the high EQ-5D ceiling effects (58% of participants obtained a utility value of 1). Recently, research has shown high EQ-5D ceiling effects in the general population [65,66]. These findings, together with results found here, suggest that the measure has limited value in assessing the utility of the intervention in this group, especially if there is an expectation that health will improve with an intervention. Future analyses might instead explore disability adjusted life years (DALYs) or negative health events (e.g. vascular diseases) avoided. Moreover, it may be that longer term follow-ups of two to three years are necessary to fully realize the costs and benefits of workplace programs of this kind. The improvements in health outcomes demonstrated in this study, if retained, could have significant implications for quality and length of life, but it was not possible to capture these benefits here.

### Strengths and limitations

Our study reports a robust evaluation of a sustainable and flexible intervention which can be implemented across a range of organizations without the need for specialist expertise. However, the necessity of evaluating an intervention with 'real-world' application combined with the constraints of conducting a rigorous analysis resulted in some tensions. These are described below.

In terms of measurement it may be that our sample were subject to 'selection' bias, where by more active individuals explicitly volunteered to be part of the study (although, within our sample the proportion of individuals meeting the recommended guidelines of 150 minutes of at least moderate intensity physical activity at baseline, was in line with population estimates at 40%). This may limit generalizability of our findings.

There were also issues in relation to the implementation of the intervention. Although randomization is considered the gold-standard for this type of effectiveness research it does have drawbacks and recently researchers have begun to question this design for organizational interventions [67,68]. The most obvious is that it creates an artificial situation that does not reflect the way that organizations usually work to effect change. In the study reported here there was tension between the need to maximise the recruitment and retention of participants within teams and the need to provide a fair test of the intervention. It is important for funders to be aware that the constraints of rigorous evaluation can mean that organizations behave in a different way to that which they would in the real world. For example, although managers within our bus company worksites were initially keen to participate in the study and worksites were randomized to control and intervention groups, attracting enthusiastic facilitators and encouraging participation amongst employees was more challenging. This meant that the quality of the intervention delivery and willingness to participate was low. Although, generally fidelity of delivering the components was high (6.4/8 components delivered), this tells us little about the quality of delivery [cf.] [69,70] something which anecdotal evidence from facilitators across the worksites suggested varied widely.

Feedback from facilitators also indicated that the one day training and the detailed manual meant that they were able to deliver the intervention. However, some facilitators felt less capable of dealing with the challenges of unenthusiastic team members. These difficulties were experienced most acutely in workplaces where people did not work as part of a team and where face-to-face communication with participants was difficult for the facilitators to achieve. These findings are important and point to the importance of contextual factors for the successful delivery of the intervention. We plan to systematically explore the impact of quality of delivery on our outcomes and to report our findings elsewhere.

This intervention was designed to tackle awareness, motivation and environment. Whilst the first two components were adequately addressed, the environmental prompts and letters of management support represent only weak proxies for the changes to organizational routines and work environments that might be necessary (alongside individual level interventions) to promote larger shifts in physical activity behavior amongst employees.

Despite these limitations, the current study has a number of strengths. First, we report the application of a sustainable intervention evaluated in a real-world setting. Second the study reported an evaluation using a cluster randomized controlled trial, adhering to CONSORT guidelines, including the application of multi-level modelling to ascertain the effects of the intervention controlling for similarities in individuals and behaviors dependent on type of worksite. Third, we assess physical activity and health measures nine months post-intervention. This is important because although studies may be able to demonstrate short-term effects on physical activity levels, for the purposes of promoting public health, evidence needs to support the effectiveness of such interventions for outcomes over longer follow-up periods.

## Conclusions

In conclusion, the current study tested a flexible workplace physical activity intervention in a cluster randomized controlled trial. Whilst the intervention did not impact self-reported MET minutes of physical activity, significant beneficial effects were apparent for systolic blood pressure and resting heart rate.

## Competing interests

The authors declare that they have no competing interests.

## Authors' contributions

RM and RL conducted the study and drafted the manuscript. RM, RL, MC and CJ contributed to the design of the study and commented on drafts of the manuscript. DM performed the health economic analysis and contributed to drafting of the manuscript. RW supported the statistical analysis and commented on drafts of the manuscript. All authors read and approved the final manuscript.

## Supplementary Material

Additional file 1 **Suggested timetable**. Click here for file

Additional file 2 **Behavior change techniques**. Click here for file

Additional file 3 **Cluster characteristics at baseline**. Click here for file

Additional file 4 **Outcome measures at baseline**. Click here for file
