# Supplementary material for: Testing a workplace physical activity intervention: a cluster randomized controlled trial
Source: Int J Behav Nutr Phys Act. 2011 Apr 11;8:29. doi: 10.1186/1479-5868-8-29 (PMC3094266; doi:10.1186/1479-5868-8-29)
Supplement: Additional file 1 — Suggested timetable. [file 1479-5868-8-29-S1.DOC]

# Suggested timetable: AME FOR ACTIVITY INTERVENTION

Enter launch date here: This will be week one of the intervention

| Key Tasks | AME material | TICK & date when done | Month one | | | | Month two | | | | Month three | | | |
| --- | --- | --- | --- | --- | --- | --- | --- | --- | --- | --- | --- | --- | --- | --- |
|  |  |  | wk1 (launch) | wk2 | wk3 | wk4 | wk5 | wk6 | wk7 | wk8 | wk9 | wk10 | wk11 | wk12 |
| Distributing leaflets | 1. AME for health |  | * |  |  |  |  |  |  |  |  |  |  |  |
|  | 2. AME for wellbeing |  |  |  |  |  | * |  |  |  |  |  |  |  |
|  | 3. AME for life |  |  |  |  |  |  |  |  |  | * |  |  |  |
| Distributing monitoring tool | AME: Keeping track magnet |  | * |  |  |  |  |  |  |  |  |  |  |  |
| Putting up posters in visible locations | Poster 1: AME for health |  | * till wk 5 |  |  |  |  |  |  |  |  |  |  |  |
|  | Poster 2: AME for wellbeing |  |  |  |  |  | * till wk 9 |  |  |  |  |  |  |  |
|  | Poster 3: AME for life |  |  |  |  |  |  |  |  |  | *till wk12 |  |  |  |
|  | Poster 4: Plan to make it happen |  | * till wk 7 |  |  |  |  |  |  |  |  |  |  |  |
|  | Poster 5: Two can make it happen |  |  |  |  |  |  |  | * till wk 12 |  |  |  |  |  |
|  | Poster 6: BHF |  | * till wk 5 |  |  |  |  |  |  |  |  |  |  |  |
|  | Poster 7: BHF |  |  |  |  |  | * till wk 9 |  |  |  |  |  |  |  |
|  | Poster 8: BHF |  |  |  |  |  |  |  |  |  | * till wk 12 |  |  |  |
| Distributing letters of management support | Letter 1 |  | * |  |  |  |  |  |  |  |  |  |  |  |
|  | Letter 2 |  |  |  |  |  |  |  |  |  |  |  |  | * |
| Running a quiz | Knowledge quiz |  | * |  |  |  |  |  |  |  |  |  |  |  |
| Running team challenges | Team Challenge (decided by facilitator) |  |  | * |  |  |  | * |  |  |  | * |  |  |
| Distributing reminders | Reminder 1 |  |  |  | * |  |  |  |  |  |  |  |  |  |
|  | Reminder 2 |  |  |  |  |  |  |  | * |  |  |  |  |  |
|  | Reminder 3 |  |  |  |  |  |  |  |  |  |  |  | * |  |
| Personalising and distributing newsletters | Newsletter 1 |  |  |  |  | * |  |  |  |  |  |  |  |  |
|  | Newsletter 2 |  |  |  |  |  |  |  |  | * |  |  |  |  |
|  | Newsletter 3 |  |  |  |  |  |  |  |  |  |  |  |  | * |
