# Supplementary material for: Testing a workplace physical activity intervention: a cluster randomized controlled trial
Source: Int J Behav Nutr Phys Act. 2011 Apr 11;8:29. doi: 10.1186/1479-5868-8-29 (PMC3094266; doi:10.1186/1479-5868-8-29)
Supplement: Additional file 2 — Behavior change techniques. [file 1479-5868-8-29-S2.DOC]

# ADDITIONAL FILE TWO: Behaviour change techniques used in AME for ACTIVITY intervention[[1]](#footnote-2)

| *Technique* | *Quiz* | *Leaflet* | *Poster* | *Monitor* | *Mgt Letter* | *Newsletter* | *Reminder* | *Team challenge* |
| --- | --- | --- | --- | --- | --- | --- | --- | --- |
| 1. Info health | X | X | X | X | X | X | X | X |
| 2. Info consequences | X | X | X |  | X | X | X |  |
| 3. Info. approval |  |  |  |  | X | X |  |  |
| 4. Form intention |  | X |  | X |  | X | X | X |
| 5. Barrier | X | X |  |  |  | X |  | X |
| 6. Encourage |  |  |  |  |  |  |  |  |
| 7. Graded tasks |  |  |  |  |  | X |  |  |
| 8. Instruction |  | X |  |  |  | X | X |  |
| 9. Model |  |  |  |  |  |  |  |  |
| 10. Specific goal setting |  | X | X | X |  |  |  | X |
| 11. Review of b’ral goals |  | X |  | X |  |  |  | X |
| 12. Self-monitor |  | X |  | X |  |  |  | X |
| 13. Feedback |  | X |  |  |  |  |  | X |
| 14. Rewards |  | X |  | X |  | X |  | X |
| 15. Prompts or cues |  | X | X |  |  | X |  | X |
| 16. Behavioural contract |  | X |  |  |  |  |  |  |
| 17. Practice |  |  |  |  |  |  |  |  |
| 18. Follow-up prompts |  |  |  |  |  |  |  |  |
| 19. Social comparison |  | X |  |  |  | X | X | X |
| 20. Social support/ social change | X | X | X |  | X | X | X | X |
| 21. Role model |  | X |  |  |  |  |  |  |
| 22. Self-talk |  |  |  |  |  |  |  |  |
| 23. Relapse prevention |  |  |  |  |  |  |  |  |
| 24. Stress management |  |  |  |  |  |  |  |  |
| 25. Motivational interviewing |  |  |  |  |  |  |  |  |
| 26. Time management |  | X | X | X | X | X |  |  |

Key: Info. Information. A full list of these techniques can be found in Abraham and Michie (2008).

X = technique used in intervention materials. Note: The intervention materials were also coded against 10 further techniques but none were identified as present. These techniques included: provide normative information about other’s behaviour, prompt anticipated regret, fear arousal, prompting focus on past success, emotional control training, general communication skills, use of imagery, environmental restricting, shaping and prompting generalisation of target behaviour.

1. [↑](#footnote-ref-2)
