# Supplementary material for: Testing a workplace physical activity intervention: a cluster randomized controlled trial
Source: Int J Behav Nutr Phys Act. 2011 Apr 11;8:29. doi: 10.1186/1479-5868-8-29 (PMC3094266; doi:10.1186/1479-5868-8-29)
Supplement: Additional file 3 — Cluster characteristics at baseline. [file 1479-5868-8-29-S3.DOC]

Additional file 3. Sample characteristics by cluster

| Organisation | Matched pair | N | | Mean Age | | N Male | | N Man /Prof occ.^a^ | | N mean health sc.^b^ | | Mean baseline mod+vig MET minutes / Week (Standard dev.)^c^ | | N baseline meet rec.^d^ | |
| --- | --- | --- | --- | --- | --- | --- | --- | --- | --- | --- | --- | --- | --- | --- | --- |
|  |  | C | I | C | I | C | I | C | I | C | I | C | I | C | I |
| Local government organisation | 1 | 51 | 32 | 43 | 38 | 26 | 9 | 30 | 24 | 71 | 66 | 1447 (2027) | 762  (1129) | 21 | 7 |
|  | 2 | 23 | 25 | 40 | 44 | 13 | 6 | 19 | 14 | 64 | 64 | 828 (1489) | 736 (835) | 11 | 10 |
|  | 3 | 19 | 29 | 41 | 40 | 5 | 9 | 13 | 16 | 73 | 69 | 1091 (1996) | 1468 (2363) | 5 | 8 |
|  | 4 | 33 | 32 | 43 | 47 | 7 | 6 | 21 | 21 | 69 | 66 | 1243 (2065) | 1339 (1543) | 12 | 14 |
|  | 5 | 10 | 8 | 34 | 43 | 2 | 1 | 8 | 4 | 68 | 78 | 460 (458) | 210 (247) | 0 | 2 |
|  | 6 | 22 | 32 | 43 | 42 | 7 | 7 | 13 | 22 | 75 | 70 | 987 (1019) | 1144 (1829) | 9 | 9 |
|  | 7 | 14 | 13 | 44 | 39 | 4 | 2 | 9 | 7 | 72 | 71 | 447 (388) | 716 (707) | 3 | 4 |
|  | 8 | 30 | 59 | 45 | 42 | 21 | 22 | 20 | 36 | 76 | 72 | 1252 (1232) | 1127 (1395) | 13 | 19 |
|  | 9 | 18 | 9 | 40 | 43 | 11 | 2 | 14 | 5 | 68 | 62 | 889 (1255) | 615 (850) | 5 | 2 |
|  | 10 | 33 | 22 | 43 | 46 | 4 | 12 | 25 | 15 | 64 | 70 | 697 (904) | 1024 (875) | 15 | 11 |
|  | **Overall** | **253** | **261** | **42** | **42** | **100** | **76** | **172** | **165** | **70** | **69** | **1064 (1582)** | **1039 (1456)** | **94** | **86** |
| Hospital | 11 | 18 | 14 | 36 | 38 | 0 | 8 | 17 | 13 | 78 | 69 | 861 (972) | 643 (773) | 11 | 8 |
|  | 12 | 8 | 23 | 41 | 42 | 2 | 3 | 7 | 11 | 75 | 80 | 1555 (1600) | 1275 (1051) | 3 | 7 |
|  | 13 | 23 | 6 | 47 | 42 | 1 | 3 | 1 | 0 | 69 | 63 | 2311 (3436) | 880 (1321) | 6 | 0 |
|  | 14 | 25 | 32 | 42 | 43 | 4 | 9 | 6 | 17 | 67 | 74 | 524 (739) | 1177 (1767) | 5 | 12 |
|  | 15 | 13 | 21 | 38 | 40 | 4 | 6 | 7 | 16 | 78 | 67 | 2235 (5218) | 424 (724) | 7 | 6 |
|  | 16 | 11 | 22 | 48 | 48 | 4 | 4 | 3 | 6 | 63 | 60 | 840 (1684) | 405 (1108) | 4 | 4 |
|  | 17 | 12 | 20 | 42 | 46 | 3 | 2 | 8 | 16 | 72 | 65 | 926 (656) | 916 (1186) | 2 | 9 |
|  | **Overall** | **110** | **138** | **42** | **43** | **18** | **35** | **49** | **79** | **72** | **69** | **1161 (2245)** | **832 (1232)** | **38** | **46** |
| Bus company | 18 | 70 | 85 | 45 | 46 | 61 | 77 | 3 | 3 | 68 | 66 | 649 (1184) | 670 (1286) | 20 | 18 |
|  | 19 | 37 | 40 | 47 | 46 | 34 | 32 | 0 | 2 | 58 | 71 | 747 (1307) | 918 (1295) | 12 | 24 |
|  | **Overall** | **107** | **125** | **46** | **46** | **95** | **109** | **3** | **5** | **63** | **69** | **682 (1219)** | **747 (1287)** | **32** | **46** |
| National Government Organisation | **20** | **83** | **89** | **38** | **43** | **44** | **55** | **76** | **70** | **69** | **68** | **1114 (1502)** | **1351 (1966)** | **33** | **38** |
| University | 21 | 34 | 38 | 42 | 41 | 12 | 12 | 16 | 21 | 72 | 68 | 1907 (1946) | 2036 (2714) | 16 | 21 |
|  | 22 | 11 | 11 | 54 | 47 | 9 | 9 | 2 | 3 | 72 | 75 | 3980 (3338) | 2851 (2668) | 7 | 7 |
|  | **Overall** | **45** | **49** | **45** | **42** | **21** | **21** | **18** | **24** | **72** | **70** | **2321 (2390)** | **2235 (2695)** | **23** | **28** |

Key: C: control; I: intervention.

^a^ Numbers classed as managerial / professional occupations

^b^ Mean health score (0-100; higher scores=better health)

^c^ Mean baseline moderate and vigorous MET minutes a week

^d^ Numbers meeting recommended guidelines at baseline
