# Supplementary material for: Testing a workplace physical activity intervention: a cluster randomized controlled trial
Source: Int J Behav Nutr Phys Act. 2011 Apr 11;8:29. doi: 10.1186/1479-5868-8-29 (PMC3094266; doi:10.1186/1479-5868-8-29)
Supplement: Additional file 4 — Outcome measures at baseline. [file 1479-5868-8-29-S4.DOC]

Additional file 4 Outcome measures by time point (NOTE – these values not adjusted for cluster effects or effects of control variables such as gender, age, health status and season of measurement).

| Outcome measure |  | Baseline | Time 2 | Time 3 | Time 4 |
| --- | --- | --- | --- | --- | --- |
| MET minutes moderate / vigorous | Control | 1124.02 (1753.51)  N=485 | 1242.44 (1797.54)  N=416 | 1280.93 (1855.42)  N=365 | 1266.74 (1652.95)  N=378 |
|  | Intervention | 1098.80 (1662.08)  N=548 | 1441.49 (1764.52)  N=422 | 1349.99 (1770.43)  N=378 | 1328.43 (1857.27)  N=385 |
| Systolic blood pressure | Control | 122.67 (15.69)  N=575 |  |  | 118.86 (14.87)  N=310 |
|  | Intervention | 123.24  (16.10)  N=639 |  |  | 118.73 (14.31)  N=300 |
| Diastolic blood pressure | Control | 79.57 (10.31)  N=575 |  |  | 77.41 (9.90)  N=310 |
|  | Intervention | 79.54 (10.68)  N=639 |  |  | 77.39 (9.24)  N=300 |
| Resting heart rate | Control | 71.14 (69.53)  N=572 |  |  | 69.53 (10.63)  N=307 |
|  | Intervention | 71.62 (11.27)  N=633 |  |  | 68.23 (9.75)  N=298 |
| Percentage body fat | Control | 31.36 (7.69)  N=574 |  |  | 30.75 (7.83)  N=311 |
|  | Intervention | 31.74 (7.83) N=629 |  |  | 31.24 (7.69) N=298 |
| Body mass index | Control | 25.96 (4.67)  N=575 |  |  | 25.33 (4.06)  N=311 |
|  | Intervention | 26.18 (5.20)  N=638 |  |  | 25.38 (4.59)  N=301 |
